# Chlorido[*N*′-(2-oxidobenzil­idene)acetohydrazide-κ^2^ *O*,*N*′,*O*′]copper(II) dihydrate

**DOI:** 10.1107/S1600536809000105

**Published:** 2009-01-08

**Authors:** Farba Bouyagui Tamboura, Mohamed Gaye, Abdou Salam Sall, Aliou Hamady Barry, Youssouph Bah

**Affiliations:** a Département de Chimie, Faculté des Sciences et Techniques, Université Cheikh Anta Diop, Dakar, Senegal; b Département de Chimie, Faculté des Sciences, Université de Nouakchott, Nouakchott, Mauritania; c Département de Chimie, Faculté des Sciences, Université de Conakry, Conakry, Guinea

## Abstract

In the title complex, [Cu(C_9_H_9_N_2_O_2_)Cl]·2H_2_O, prepared from the Schiff base ligand *N*′-(2-hydroxy­benzil­idene)aceto­hydrazide and copper(II) chloride, the Cu^II^ atom is coord­inated by two O atoms and one N atom from the ligand and by a Cl atom in a distorted square-planar geometry. The two donor O atoms of the tridentate Schiff base ligand are in a *trans* arrangement. In the crystal structure, there is an extensive inter­molecular hydrogen-bonding network; N—H⋯O, O—H⋯O and O—H⋯Cl inter­actions, involving the uncoordinated water mol­ecules, lead to the formation of a two-dimensional network parallel to the *ab* plane.

## Related literature

For related structures, see: Ainscough *et al.* (1998 ▶); Chan *et al.* (1995 ▶); Koh *et al.* (1998 ▶). For similar square-planar copper(II) complexes, see: Li *et al.* (2008 ▶); Qiu & Wu (2004 ▶).
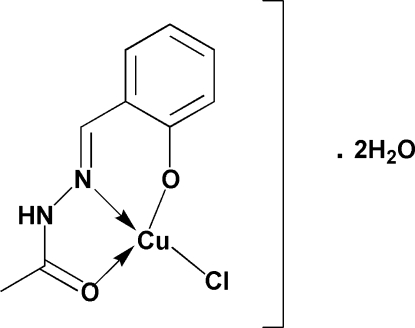


## Experimental

### 

#### Crystal data

[Cu(C_9_H_9_N_2_O_2_)Cl]·2H_2_O
*M* *_r_* = 312.20
Triclinic, 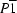

*a* = 6.762 (2) Å
*b* = 8.987 (2) Å
*c* = 10.312 (3) Å
α = 76.940 (11)°
β = 84.645 (12)°
γ = 81.903 (13)°
*V* = 603.1 (3) Å^3^
*Z* = 2
Mo *K*α radiation
μ = 2.04 mm^−1^
*T* = 173 (2) K
0.16 × 0.12 × 0.10 mm

#### Data collection

Nonius KappaCCD diffractometer
Absorption correction: none
5193 measured reflections
3520 independent reflections
3036 reflections with *I* > 2σ(*I*)
*R*_int_ = 0.021

#### Refinement

*R*[*F* ^2^ > 2σ(*F* ^2^)] = 0.032
*wR*(*F* ^2^) = 0.075
*S* = 1.06
3520 reflections
174 parameters
4 restraints
H atoms treated by a mixture of independent and constrained refinement
Δρ_max_ = 0.50 e Å^−3^
Δρ_min_ = −0.58 e Å^−3^

### 

Data collection: *COLLECT* (Nonius, 1998 ▶); cell refinement: *DENZO*/*SCALEPACK* (Otwinowski & Minor, 1997 ▶); data reduction: *DENZO*/*SCALEPACK*; program(s) used to solve structure: *SHELXS97* (Sheldrick, 2008 ▶); program(s) used to refine structure: *SHELXL97* (Sheldrick, 2008 ▶); molecular graphics: *PLATON* (Spek, 2003 ▶); software used to prepare material for publication: *SHELXL97*.

## Supplementary Material

Crystal structure: contains datablocks I, global. DOI: 10.1107/S1600536809000105/su2089sup1.cif

Structure factors: contains datablocks I. DOI: 10.1107/S1600536809000105/su2089Isup2.hkl

Additional supplementary materials:  crystallographic information; 3D view; checkCIF report

## Figures and Tables

**Table:** 

Cu1—O1	1.8951 (13)
Cu1—N2	1.9373 (15)
Cu1—O2	1.9628 (13)
Cu1—Cl1	2.2203 (5)

**Table:** 

O1—Cu1—N2	92.17 (6)
O1—Cu1—O2	170.21 (6)
N2—Cu1—O2	81.39 (6)
O1—Cu1—Cl1	93.63 (4)
N2—Cu1—Cl1	173.31 (5)
O2—Cu1—Cl1	93.27 (4)

**Table 2:** Hydrogen-bond geometry (Å, °)

*D*—H⋯*A*	*D*—H	H⋯*A*	*D*⋯*A*	*D*—H⋯*A*
N1—H1⋯O3	0.81 (3)	1.88 (3)	2.683 (2)	177 (3)
O3—H*W*1⋯O4^i^	0.837 (17)	1.941 (18)	2.777 (3)	177 (3)
O3—H*W*2⋯Cl1^ii^	0.837 (18)	2.41 (2)	3.2333 (18)	168 (4)
O4—H*W*3⋯O1^iii^	0.840 (18)	2.087 (19)	2.916 (2)	169 (3)
O4—H*W*4⋯O1	0.835 (18)	2.43 (2)	3.191 (2)	153 (3)
O4—H*W*4⋯Cl1	0.835 (18)	2.80 (3)	3.4648 (17)	137 (3)

Symmetry codes: (i) 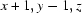; (ii) 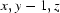; (iii) 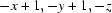.

**Fig. 1.**
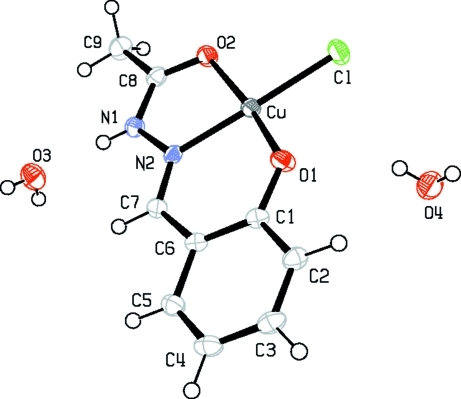
A view of the molecular structure of compound (I), showing the atom-numbering scheme. Displacement ellipsoids are drawn at the 50% probability level.

**Fig. 2.**
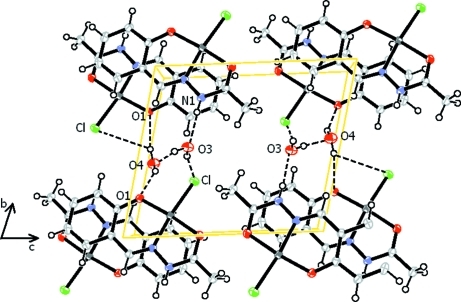
A view along the a axis of the crystal packing of compound (I), showing the N—H···O, O—H···O and O—H···Cl hydrogen bonds as dashed lines (see Table 2 for details).

## supplementary crystallographic information

### Comment

The title complex, (I), was prepared by the reaction of the Schiff base ligand *N*'-(2-hydroxybenzilidene)acetohydrazide with copper(II) chloride. The molecular structure of (I) is illustrated in Fig. 1, and selected bond distances and angles are given in Table 1. Atom Cu1 is coordinated to two O-atoms and one N-atom from the Schiff base ligand and to one chloride atom. The Cu1-N and Cu1-O bond distances are similar to those observed in other Cu^II^ complexes of the same and similar tridentate ligands (Ainscough *et al.*,  1998; Chan *et al.*,  1995; Koh *et al.*,  1998). The Cu1-Cl distance (2.2203 (5) Å) is similar to that observed in other copper(II) square-planar complexes (Li *et al.*,  2008; Qiu & Wu, 2004).  The two O donor atoms are in a trans arrangement with a O-Cu1-O angle of 170.21 (6)°.  The angles around atom Cu1 are in the range of 81.39 (6) - 173.31 (5)°. The sum of the angles around atom Cu1 is 360.46°, suggesting that the geometry around the copper atom is distorted square-planar.

In the crystal structure of (I) there is an extensive intermolecular hydrogen bonding network (Fig. 2). N—H···O, O—H···O and O—H···Cl interactions (Table 2), involving the lattice water molecules, lead to the formation of a two-dimensional network parallel to the ab plane.

### Experimental

To 0.356 g (2.0 mmol) of *N*'-(2-hydroxybenzilidene)acetohydrazide in 20 ml of ethanol was added a solution of copper chloride dihydrate (0.341 g, 2 mmol) in 10 ml of ethanol. The resulting mixture was refluxed for 1 h. After cooling the resulting solution was filtered, and the filtrate left for slow evaporation. Small green crystals of compound (I), suitable for X-ray analysis, was obtained in good yield (0.600 g; 96.0 %). IR (cm^-1^,KBr): 3490, 1675, 1640, 1620, 1580, 11570, 1465. UV (nm): 720, 600, 400. µ_eff_ = 1.80 µ_B_. Conductance: Λ=13 S cm^2^ mol^-1^. Analysis calculated for C_9_H_13_ClCuN_2_O_4_: C 34.62, H 4.20, N 8.97 %; found: C 34.60, H 4.18, N 8.65 %.

### Refinement

The NH hydrogen atom was located in a difference Fourier map and freely refined: 0.81 (3) Å. The water H-atoms were located in difference Fourier maps and refined isotropically with the O-H distances retrainted to 0.88 (2) Å. The remainder of the H-atoms were placed in calculated positions and treated as riding atoms: C-H = 0.95 - 0.98 Å with *U*_iso_(H) = 1.2 or 1.5*U*_eq_(parent C-atom).

### Crystal data

**Table:**

[Cu(C_9_H_9_N_2_O_2_)Cl]·2H_2_O	*Z* = 2
*M**_r_* = 312.20	*F*(000) = 318
Triclinic, *P*1	*D*_x_ = 1.719 Mg m^−^^3^
Hall symbol: -P 1	Mo *K*α radiation, λ = 0.71073 Å
*a* = 6.762 (2) Å	Cell parameters from 2138 reflections
*b* = 8.987 (2) Å	θ = 1.0–30.0°
*c* = 10.312 (3) Å	µ = 2.04 mm^−^^1^
α = 76.940 (11)°	*T* = 173 K
β = 84.645 (12)°	Prism, green
γ = 81.903 (13)°	0.16 × 0.12 × 0.10 mm
*V* = 603.1 (3) Å^3^	

### Data collection

**Table:**

Nonius KappaCCD diffractometer	3036 reflections with *I* > 2σ(*I*)
Radiation source: fine-focus sealed tube	*R*_int_ = 0.021
graphite	θ_max_ = 30.0°, θ_min_ = 2.8°
π[Please check] scans	*h* = −9→8
5193 measured reflections	*k* = −12→12
3520 independent reflections	*l* = −13→14

### Refinement

**Table:**

Refinement on *F*^2^	Primary atom site location: structure-invariant direct methods
Least-squares matrix: full	Secondary atom site location: difference Fourier map
*R*[*F*^2^ > 2σ(*F*^2^)] = 0.032	Hydrogen site location: inferred from neighbouring sites
*wR*(*F*^2^) = 0.075	H atoms treated by a mixture of independent and constrained refinement
*S* = 1.06	*w* = 1/[s^2^(*F*_o_^2^) + (0.0139*P*)^2^ + 0.4006*P*] where *P* = (*F*_o_^2^ + 2*F*_c_^2^)/3
3520 reflections	(Δ/σ)_max_ = 0.036
174 parameters	Δρ_max_ = 0.50 e Å^−^^3^
4 restraints	Δρ_min_ = −0.58 e Å^−^^3^

### Special details

**Table:**

Geometry. All e.s.d.'s (except the e.s.d. in the dihedral angle between two l.s. planes) are estimated using the full covariance matrix. The cell e.s.d.'s are taken into account individually in the estimation of e.s.d.'s in distances, angles and torsion angles; correlations between e.s.d.'s in cell parameters are only used when they are defined by crystal symmetry. An approximate (isotropic) treatment of cell e.s.d.'s is used for estimating e.s.d.'s involving l.s. planes.
Refinement. Refinement of *F*^2^ against ALL reflections. The weighted *R*-factor *wR* and goodness of fit *S* are based on *F*^2^, conventional *R*-factors *R* are based on *F*, with *F* set to zero for negative *F*^2^. The threshold expression of *F*^2^ > σ(*F*^2^) is used only for calculating *R*-factors(gt) *etc*. and is not relevant to the choice of reflections for refinement. *R*-factors based on *F*^2^ are statistically about twice as large as those based on *F*, and *R*- factors based on ALL data will be even larger.

### Fractional atomic coordinates and isotropic or equivalent isotropic displacement parameters (Å^2^)

**Table:**

	*x*	*y*	*z*	*U*_iso_*/*U*_eq_	
Cu1	0.70935 (3)	0.12114 (2)	0.20663 (2)	0.01876 (7)	
Cl1	0.59285 (8)	0.32377 (5)	0.29307 (5)	0.02773 (12)	
O1	0.6694 (2)	0.22971 (14)	0.02897 (14)	0.0247 (3)	
O2	0.7955 (2)	−0.00478 (15)	0.37823 (14)	0.0238 (3)	
O3	0.9689 (3)	−0.47969 (18)	0.23882 (18)	0.0330 (4)	
O4	0.2576 (3)	0.44891 (19)	0.04512 (18)	0.0342 (4)	
N1	0.8652 (2)	−0.18532 (18)	0.25675 (17)	0.0200 (3)	
H1	0.899 (4)	−0.273 (3)	0.249 (3)	0.032 (7)*	
N2	0.7995 (2)	−0.07024 (16)	0.15054 (15)	0.0169 (3)	
C1	0.6714 (3)	0.1693 (2)	−0.07799 (19)	0.0195 (4)	
C2	0.6135 (3)	0.2688 (2)	−0.1980 (2)	0.0229 (4)	
H2	0.5741	0.3749	−0.1995	0.050*	
C3	0.6125 (3)	0.2164 (2)	−0.3137 (2)	0.0251 (4)	
H3	0.5735	0.2867	−0.3934	0.050*	
C4	0.6682 (3)	0.0608 (2)	−0.3152 (2)	0.0263 (4)	
H4	0.6668	0.0249	−0.3949	0.050*	
C5	0.7249 (3)	−0.0390 (2)	−0.1993 (2)	0.0231 (4)	
H5	0.7630	−0.1448	−0.1998	0.050*	
C6	0.7283 (3)	0.0114 (2)	−0.07873 (19)	0.0189 (4)	
C7	0.7920 (3)	−0.1029 (2)	0.03622 (19)	0.0193 (4)	
H7	0.8299	−0.2062	0.0273	0.050*	
C8	0.8581 (3)	−0.1417 (2)	0.37220 (19)	0.0210 (4)	
C9	0.9213 (3)	−0.2590 (2)	0.4927 (2)	0.0286 (4)	
H9A	0.9653	−0.3581	0.4679	0.050*	
H9B	0.8083	−0.2698	0.5595	0.050*	
H9C	1.0320	−0.2262	0.5298	0.050*	
HW1	1.054 (4)	−0.504 (3)	0.180 (2)	0.050 (9)*	
HW2	0.873 (4)	−0.529 (4)	0.240 (4)	0.089 (13)*	
HW3	0.286 (5)	0.539 (2)	0.014 (3)	0.067 (10)*	
HW4	0.367 (4)	0.398 (4)	0.067 (4)	0.076 (11)*	

### Atomic displacement parameters (Å^2^)

**Table:**

	*U*^11^	*U*^22^	*U*^33^	*U*^12^	*U*^13^	*U*^23^
Cu1	0.02308 (13)	0.01631 (12)	0.01686 (13)	−0.00064 (8)	−0.00474 (9)	−0.00321 (8)
Cl1	0.0375 (3)	0.0199 (2)	0.0257 (3)	0.00195 (19)	−0.0029 (2)	−0.00765 (18)
O1	0.0400 (8)	0.0171 (6)	0.0174 (7)	−0.0032 (6)	−0.0073 (6)	−0.0024 (5)
O2	0.0316 (8)	0.0215 (6)	0.0178 (7)	0.0027 (5)	−0.0065 (6)	−0.0049 (5)
O3	0.0398 (10)	0.0234 (7)	0.0359 (10)	−0.0038 (7)	−0.0016 (8)	−0.0069 (7)
O4	0.0302 (9)	0.0296 (8)	0.0425 (10)	−0.0032 (7)	−0.0075 (7)	−0.0052 (7)
N1	0.0234 (8)	0.0161 (7)	0.0192 (8)	−0.0002 (6)	−0.0047 (6)	−0.0013 (6)
N2	0.0173 (7)	0.0153 (7)	0.0173 (8)	−0.0008 (5)	−0.0048 (6)	−0.0008 (6)
C1	0.0191 (9)	0.0223 (9)	0.0176 (9)	−0.0052 (7)	−0.0017 (7)	−0.0034 (7)
C2	0.0249 (10)	0.0219 (9)	0.0208 (10)	−0.0048 (7)	−0.0042 (8)	−0.0002 (7)
C3	0.0233 (10)	0.0341 (10)	0.0167 (9)	−0.0066 (8)	−0.0031 (8)	−0.0003 (8)
C4	0.0268 (10)	0.0367 (11)	0.0179 (10)	−0.0075 (8)	−0.0012 (8)	−0.0088 (8)
C5	0.0223 (9)	0.0263 (9)	0.0223 (10)	−0.0042 (7)	−0.0001 (8)	−0.0087 (8)
C6	0.0188 (8)	0.0220 (9)	0.0169 (9)	−0.0050 (7)	−0.0023 (7)	−0.0042 (7)
C7	0.0190 (9)	0.0190 (8)	0.0204 (9)	−0.0028 (7)	−0.0018 (7)	−0.0049 (7)
C8	0.0197 (9)	0.0242 (9)	0.0178 (9)	−0.0011 (7)	−0.0030 (7)	−0.0022 (7)
C9	0.0357 (11)	0.0271 (10)	0.0198 (10)	0.0008 (8)	−0.0066 (9)	0.0009 (8)

### Geometric parameters (Å, °)

**Table:**

Cu1—O1	1.8951 (13)	C1—C6	1.419 (3)
Cu1—N2	1.9373 (15)	C2—C3	1.380 (3)
Cu1—O2	1.9628 (13)	C2—H2	0.9500
Cu1—Cl1	2.2203 (5)	C3—C4	1.399 (3)
O1—C1	1.333 (2)	C3—H3	0.9500
O2—C8	1.257 (2)	C4—C5	1.373 (3)
O3—HW1	0.837 (17)	C4—H4	0.9500
O3—HW2	0.837 (18)	C5—C6	1.420 (3)
O4—HW3	0.840 (18)	C5—H5	0.9500
O4—HW4	0.835 (18)	C6—C7	1.438 (2)
N1—C8	1.330 (2)	C7—H7	0.9500
N1—N2	1.384 (2)	C8—C9	1.489 (3)
N1—H1	0.81 (3)	C9—H9A	0.9800
N2—C7	1.285 (2)	C9—H9B	0.9800
C1—C2	1.406 (3)	C9—H9C	0.9800
			
O1—Cu1—N2	92.17 (6)	C2—C3—H3	119.6
O1—Cu1—O2	170.21 (6)	C4—C3—H3	119.6
N2—Cu1—O2	81.39 (6)	C5—C4—C3	118.78 (18)
O1—Cu1—Cl1	93.63 (4)	C5—C4—H4	120.6
N2—Cu1—Cl1	173.31 (5)	C3—C4—H4	120.6
O2—Cu1—Cl1	93.27 (4)	C4—C5—C6	121.80 (18)
C1—O1—Cu1	126.91 (11)	C4—C5—H5	119.1
C8—O2—Cu1	112.67 (12)	C6—C5—H5	119.1
HW1—O3—HW2	107 (3)	C1—C6—C5	119.09 (17)
HW3—O4—HW4	104 (3)	C1—C6—C7	123.89 (17)
C8—N1—N2	114.80 (15)	C5—C6—C7	117.02 (17)
C8—N1—H1	123.0 (18)	N2—C7—C6	122.26 (16)
N2—N1—H1	122.1 (18)	N2—C7—H7	118.9
C7—N2—N1	119.37 (15)	C6—C7—H7	118.9
C7—N2—Cu1	129.28 (12)	O2—C8—N1	119.94 (16)
N1—N2—Cu1	111.15 (12)	O2—C8—C9	121.56 (18)
O1—C1—C2	117.80 (17)	N1—C8—C9	118.49 (17)
O1—C1—C6	124.33 (16)	C8—C9—H9A	109.5
C2—C1—C6	117.88 (17)	C8—C9—H9B	109.5
C3—C2—C1	121.67 (18)	H9A—C9—H9B	109.5
C3—C2—H2	119.2	C8—C9—H9C	109.5
C1—C2—H2	119.2	H9A—C9—H9C	109.5
C2—C3—C4	120.79 (18)	H9B—C9—H9C	109.5

### Hydrogen-bond geometry (Å, °)

**Table:**

*D*—H···*A*	*D*—H	H···*A*	*D*···*A*	*D*—H···*A*
N1—H1···O3	0.81 (3)	1.88 (3)	2.683 (2)	177 (3)
O3—HW1···O4^i^	0.84 (2)	1.94 (2)	2.777 (3)	177 (3)
O3—HW2···Cl1^ii^	0.84 (2)	2.41 (2)	3.2333 (18)	168 (4)
O4—HW3···O1^iii^	0.84 (2)	2.09 (2)	2.916 (2)	169 (3)
O4—HW4···O1	0.84 (2)	2.43 (2)	3.191 (2)	153 (3)
O4—HW4···Cl1	0.84 (2)	2.80 (3)	3.4648 (17)	137 (3)

Symmetry codes: (i) *x*+1, *y*−1, *z*; (ii) *x*, *y*−1, *z*; (iii) −*x*+1, −*y*+1, −*z*.
